# Resistance to immune checkpoint inhibitors in gastric cancer

**DOI:** 10.3389/fphar.2023.1285343

**Published:** 2023-11-13

**Authors:** Kai Liu, Shiman Yuan, Chenyu Wang, Hong Zhu

**Affiliations:** ^1^ The Clinical Medical College, Guizhou Medical University, Guiyang, China; ^2^ The Second Clinical Medical College, Lanzhou University, Lanzhou, China; ^3^ Cancer Center, Department of Medical Oncology, West China Hospital, Sichuan University, Chengdu, China

**Keywords:** immunotherapy resistance, mechanism, immune checkpoint inhibitor, gastric cancer, tumor microenvironment

## Abstract

Gastric cancer (GC) is one of the most common gastrointestinal malignancies worldwide. In the past decade, with the development of early diagnostic techniques, a clear decline in GC incidence has been observed, but its mortality remains high. The emergence of new immunotherapies such as immune checkpoint inhibitors (ICIs) has changed the treatment of GC patients to some extent. However, only a small number of patients with advanced GC have a durable response to ICI treatment, and the efficacy of ICIs is very limited. Existing studies have shown that the failure of immunotherapy is mainly related to the development of ICI resistance in patients, but the understanding of the resistance mechanism is still insufficient. Therefore, clarifying the mechanism of GC immune resistance is critical to improve its treatment and clinical benefit. In this review, we focus on summarizing the mechanisms of primary or acquired resistance to ICI immunotherapy in GC from both internal and external aspects of the tumor. At the same time, we also briefly discuss some other possible resistance mechanisms in light of current studies.

## 1 Introduction

Gastric cancer (GC) has the fourth highest mortality and fifth highest incidence globally, and the GC prevalence is usually higher in East Asia than in Europe and the US (Sung et al., 2021). The early symptoms of GC are usually less obvious. Most patients with GC are already advanced when the tumor is found in their body, and the 5-year relative survival rate is very low (Thrift and El-Serag, 2020). Currently, endoscopic resection is the optimal treatment with favourable prognosis for early GC. Nonearly operable GC is treated with surgery. The extent of surgical resection depends on tumor location, histological subtype and TNM (Tumor Node Metastasis) category. Perioperative chemotherapy and adjuvant treatment can improve the survival rate of patients to a certain extent. The first-line treatment of advanced or metastatic unresectable GC is sequential lines of chemotherapy, such as a platinum and fluoropyrimidine doublet. Currently, trastuzumab (HER2-positive patients) and nivolumab or pembrolizumab are approved targeted therapeutic agents for GC (Lordick et al., 2022).

In the last 10 years, immunotherapy has rapidly developed, especially the application of immune checkpoint inhibitors (ICIs). These drugs work by targeting specific molecules such as programmed death-1 (PD-1) or its ligand programmed death ligand-1 (PD-L1) and cytotoxic T lymphocyte-associated antigen-4 (CTLA-4) to reestablish anti-tumor responses and prevent tumor cells from evading immune surveillance. Many advanced GC patients treated with ICIs have a good respond and a significantly longer survival (Jin et al., 2022; Kaushik et al., 2022). However, in GC patients, the efficacy of single-agent immunotherapy has been unsatisfactory despite breakthroughs in PD-1 antibody research (Wang et al., 2021). Clinical trials have also confirmed that the effectiveness of targeted drugs such as CTLA-4 and PD-1 alone or in combination with other drugs is limited for patients with advanced GC, which is far less than that of melanoma, lung cancer and other tumors (Kooshkaki et al., 2020). Existing studies have shown that the failure of immunotherapy is mainly related to the development of ICI resistance in patients, but the understanding of the resistance mechanism is still insufficient (Baxter et al., 2021). Therefore, understanding the immune resistance mechanism in GC is critical to improve its treatment and clinical benefit. The ongoing clinical trials of PD-1/PD-L1 in advanced gastric cancer (Table 1).

**TABLE 1:** The ongoing clinical trials of PD-1/PD-L1 in advanced gastric cancer.

Intervention/Treatment	Phase	Target	Condition	Study size	Estimated completion date	NCT number
Durvalumab	II	PD-L1	Gastric Adenocarcinoma	107	2023	NCT03959293
Tremelimumab		CTLA-4	Gastric Cancer			
Pembrolizumab	III	PD-1	Gastric Neoplasms	732	2024	NCT03615326
Trastuzumab		HER-2	Gastroesophageal Junction Adenocarcinoma			
Pembrolizumab	III	PD-1	Gastric Cancer	1,007	2024	NCT03221426
			Gastroesophageal Junction Cancer			
Nivolumab	II	PD-1	Adenocarcinoma of the Stomach	262	2024	NCT03647969
Ipilimumab		CTLA-4	GastroEsophageal Cancer			
Nivolumab	III	PD-1	Advanced Cancer	794	2025	NCT02743494
Atezolizumab	II	PD-L1	Gastric Cancer	674	2027	NCT03421288
			Gastroesophageal Junction Adenocarcinoma			

**FIGURE 1:**
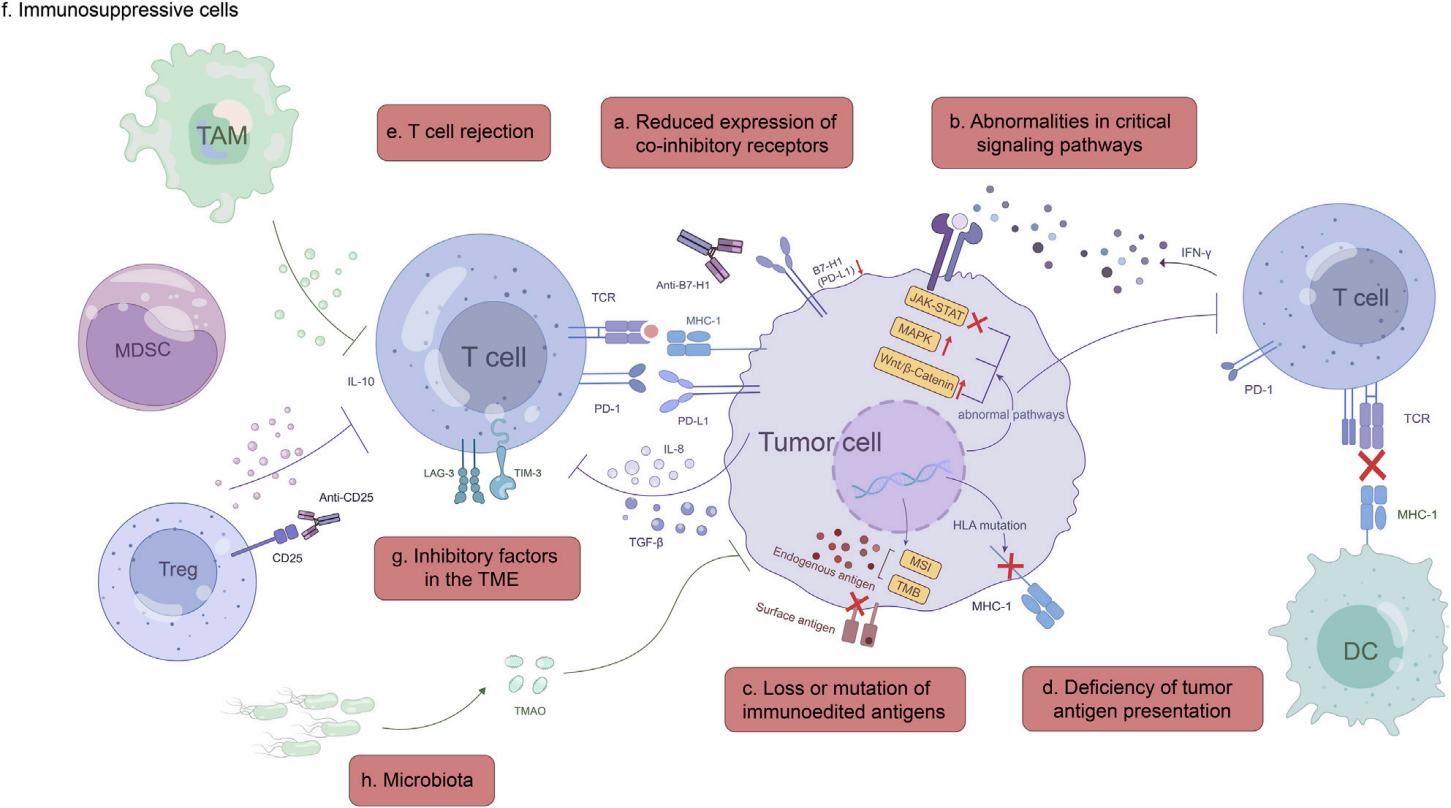
Mechanisms of ICI immunotherapy resistance. Tumor-intrinsic mechanisms: a, Reduced expression of co-inhibitory receptors, such as B7-H1(PD-L1) and B7-1; b, Abnormalities in critical signaling pathways, including IFN-γ, MAPK and Wnt/β-catenin signaling pathway; c, Loss or mutation of immunoedited antigens; d, Deficiency of tumor antigen presentation. Tumor-extrinsic mechanisms: e, T cell rejection; f, Immunosuppressive cells; g, Inhibitory factors in the TME; h, Microbiota.

In this review, we focus on the mechanisms of primary or acquired resistance to ICI immunotherapy in GC, including tumor-intrinsic, tumor-extrinsic and other mechanisms (Figure 1), and emphasize the importance of these resistance mechanisms for GC treatment in immuno-oncology.

## 2 Mechanisms of ICI immunotherapy resistance

Resistance to ICI immunotherapy can be divided into primary resistance and acquired resistance based on clinical outcomes. Primary resistance refers to an initial failure to respond to immunotherapy, whereas acquired resistance refers to disease progression after an initial response to immunotherapy. In some ways, primary and acquired resistance mechanisms overlap, which may underlie why GC develops resistance to ICIs. At present, as our research on acquired resistance is still in its infancy, in this paper, we will mainly focus on the basic and potential mechanisms of ICI treatment resistance, which are divided into tumor-intrinsic, tumor-extrinsic and other mechanisms, and do not classify them as primary or acquired to avoid complicating the problem.

### 2.1 Tumor-intrinsic mechanisms

#### 2.1.1 Reduced expression of co-inhibitory receptors

PD-1/PD-L1 inhibitors are the first group of ICIs to be used in clinical practice. The expression intensity of tumor B7-H1/PD-L1 is closely related to ICI response, so it has become a commonly used biomarker to predict the effect of ICIs (Ott et al., 2019). The combination of PD-1 and PD-L1 weakens the ability of T cells to kill tumors, inhibits T-cell receptor-mediated cytokine secretion and lymphocyte proliferation, and ultimately leads to tumor cells evading the immune system (Freeman et al., 2000). In addition, tumor-associated B7-H1 can also evade the host immune response by promoting T-cell apoptosis (Dong et al., 2002). Targeting PD-L1 with antibodies is an important strategy for GC treatment. For example, avelumab, an anti-PD-L1 mab, was shown to be well tolerated in the clinical trial in advanced GC patients and to obtain promising results in patients in some countries (Bang et al., 2018). However, according to accumulated data from several independent studies, there is still a significant proportion of cancers that express low levels of B7-H1 or even do not express B7-H1, and these patients have a poor response to anti-PD treatment (Kim et al., 2018). Thus, GC with a lack or low expression of PD-L1 is theoretically more likely to be resistant to anti-PD therapies, which is supported by the apparent decrease in PD-L1 expression levels in some GC patients (Li et al., 2020). Notably, although PD-L1 has shown important utility as a predictive biomarker in some tumor types, some patients with high levels of PD-L1 expression do not respond well to anti-PD-1 therapy, while some patients with a lack of PD-L1 expression actually do better, suggesting that apart from PD-L1, other receptors that interact with PD-1 molecules, such as PD-L2, and other factors may be involved in the response to ICIs (Yearley et al., 2017; Li et al., 2020).

CTLA-4 is a previously studied coinhibitory molecule of the B7 family, which is mainly expressed on activated T lymphocytes and introduced as a target of ICIs in the immunotherapy of tumors (Brunet et al., 1987). The interaction between B7-1/B7-2 receptors and CTLA-4 transmits signals that inhibit the activation of T lymphocytes, resulting in the inhibition of the corresponding immune response. Humanized monoclonal antibodies, such as tremelimumab and ipilimumab, inhibit the effect of CTLA-4, leading to enhanced T-cell-mediated cytotoxicity (Kooshkaki et al., 2020). In the past decade, due to their amazing antitumor efficacy and promising prospects, these anti-CTLA-4 antibodies (including other ICIs) have been accepted for the treatment of cancer, and some of them combined with other methods (such as chemotherapy and radiotherapy) have become the standard first-line therapy for some advanced cancers, such as GC, gastroesophageal cancer, and melanoma (Daud et al., 2016; Janjigian et al., 2021; Shitara et al., 2022). In general, CTLA-4 combination therapy shows enhanced antitumor efficacy, but the monotherapy of CTLA-4 inhibitors for advanced GC is still limited, and the resistance mechanism of anti-CTLA-4 treatment needs to be further studied (Jin et al., 2022). In addition, B7-1/B7-2 can interact with CD28 to transmit stimulatory signals to activate T lymphocytes. Some studies have shown that B7-1, as one of the costimulatory factors, can inhibit lymph node metastasis by enhancing immunogenicity, so the transduction of B7-1 gene may become an effective therapy for GC lymph node metastasis (Sakate et al., 2004). Compared with normal gastric tissues, the B7-1 expression is less and the mutation rate is higher in GC tissues based on the statistic data (Li et al., 2020). Accordingly, we hypothesized that the decreased expression of B7-1 in GC cells may be one of the reasons for the resistance to anti-CTLA-4 immunotherapy in GC patients.

To date, anti-PD-1/PD-L1 and anti-CTLA-4 therapy remain the two most widely used ICI immunotherapies (Kraehenbuehl et al., 2022). However, tumor cells, including GC cells has learned to evade the immune system by upregulating the expression of related receptors such as PD-L1 and PD-L2, which becomes one of their most powerful weapons against ICIs. Interestingly, the reduced or abnormal expression of related receptors in some tumor cells not only helps them evade the immune system, but also enhances their resistance to ICI treatment, which may be the result of tumor adaptation to the host immune response. Meanwhile, this undoubtedly brings great challenges to current ICI immunotherapy.

#### 2.1.2 Abnormalities in critical signaling pathways

**FIGURE 2:**
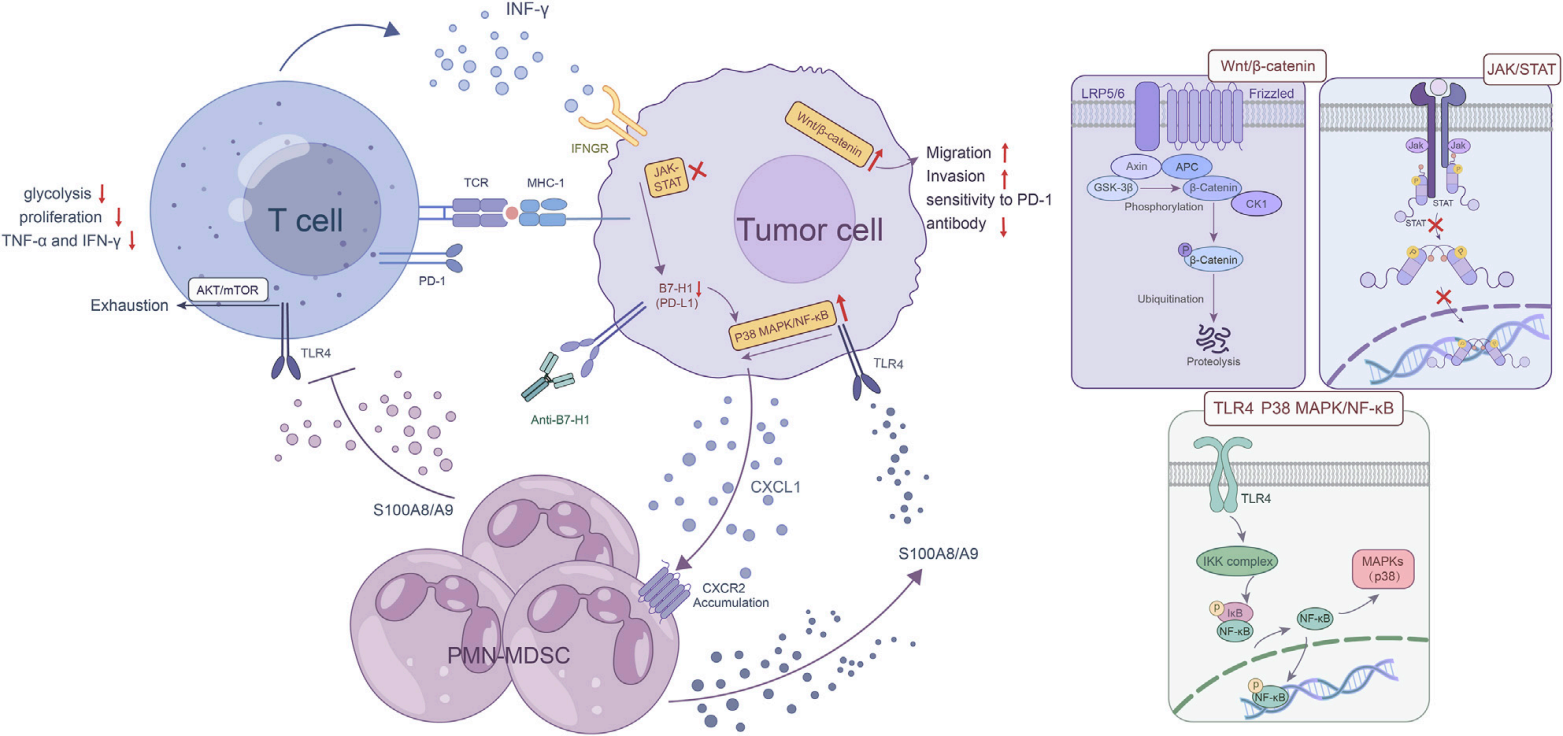
Abnormalities in critical signaling pathways. The mutation or loss of IFN-γ signaling pathway not only causes abnormal T cell function, but more importantly, the lack of PD-L1 expression in these patients, which in turn leads to ICI resistance. As a hallmark of MDSCs, the S100A8/A9 heterodimer upregulates CXCL1 expression in GC cells via the TLR4/p38 MAPK/NF-κB pathway, and CXCL1 induces the accumulation of PMN-MDSCs in GC. The activation of the S100A8/A9-TLR4/AKT/mTOR pathway eventually leads to the exhaustion CD8 (+) T cells. This exhaustion manifests as a decrease in glycolysis, proliferation, and the production of key cytokines such as TNF-α and IFN-γ and directly reduces the effectiveness of ICI treatment. In some cases, B7-H1 deficiency or binding to certain antibodies leads to increased activation of p38 MAPK, which induces apoptosis in T cells, thus reducing immunotherapy efficacy. The aberrant Wnt/β-catenin pathway activation in GC cells promotes their invasion and migration abilities, but reduces the sensitivity to PD-1 antibody.

In GC, ICI immunotherapy resistance involves the transduction of multiple signaling pathways and the complex interactions associated with them, especially certain crucial signaling pathways, such as the IFN-γ signaling, the mitogen-activated protein kinase (MAPK) pathway, and the Wnt/β-catenin pathway. Aberrant alterations in these pathways usually affect the expression of immune checkpoint molecules, which weakens T-cell recruitment and function (Figure 2). IFN-γ signaling can upregulate the expression of related cytokines and costimulatory factors in antigen presenting cells (APCs) and thus enhancing the presentation of tumor-associated antigens (TAAs). Moreover, IFN-γ triggers numerous signals in T cells, facilitating their optimal functioning. The lack of IFN-γ signaling pathways in T cells hampers T-cell responses, thereby promoting tumor growth and invasion (Ni and Lu, 2018). Recently, Mimura et al. found that PD-L1 expression is mainly controlled by JAK-STAT pathway-associated IFN-γ in GC, and clinical GC samples with PD-L1 expression are strongly positively correlated with CD8 (+) T cells in the stroma as well as IFN-γ expression (Mimura et al., 2018). Therefore, the mutation or deficiency of the IFN-γ signaling pathway not only causes abnormal T-cell function but also the lack of PD-L1 expression in these patients, which eventually contributes to ICI resistance.

The MAPK pathway, including the canonical RAS-RAF-MEK-ERK signaling cascade, plays an important role in regulation of physiological processes such as cell proliferation, differentiation, survival, and death by sending upstream signals to downstream effectors (Liu et al., 2018). The accumulation of myeloid-derived suppressor cells (MDSCs) in a variety of tumors is associated with ICI resistance. As a hallmark of MDSCs, the S100A8/A9 heterodimer upregulates CXCL1 expression in GC cells via the TLR4/p38 MAPK/NF-κB pathway, and CXCL1 induces the accumulation of PMN-MDSCs in GC. Moreover, the activation of the S100A8/A9-TLR4/AKT/mTOR pathway eventually leads to the exhaustion CD8 (+) T cells. This exhaustion manifests as a decrease in glycolysis, proliferation, and the production of key cytokines such as TNF-α and IFN-γ and directly reduces the effectiveness of ICI treatment. Furthermore, this study also showed that the accumulation of PMN-MDSCs was diminished by inhibiting CXCR2, leading to an enhanced immune response against tumors and increased sensitivity to anti-PD-1 therapy in GC cells. This preclinical study provides a crucial basis for the development of the combined therapeutic approach (Zhou et al., 2022). The study by D'Souza et al. showed that the Erk MAPK pathway plays an important role in the proliferation of activated CD8 T cells (D'Souza et al., 2008). According to another investigation, circMAPK1 expression was found to be lower in GC tissues compared to the neighboring healthy tissues. CircMAPK1 can inhibit the growth and infiltration of GC cells by encoding protein MAPK1-109aa. The protein, acting as a potent suppressor of tumors, competitively associates with MEK1 to hinder the phosphorylation of MAPK1, which ultimately leads to the inhibition of its subsequent downstream factors in the MAPK pathway (Jiang et al., 2021). In addition, compared with the typical B7-H1 blocking antibody, the antitumor effect of B7-H1 antibodies that are able to activate p38 MAPK is compromised due to the elimination of B7-H1 tumor-responsive CD8 T cells through the p38 MAPK pathway. In some cases, B7-H1 deficiency or binding to specific antibody leads to increased activation of p38 MAPK and induces apoptosis in T cells, thus reducing immunotherapy efficacy (Liu et al., 2016). Taken together, these results reveal that GC cells with aberrant MAPK pathways may be more resistant to ICI treatment, but more work is needed to determine how abnormalities in the MAPK pathway contribute to ICI therapy resistance.

Dysregulation of the Wnt/β-catenin signaling pathway is strongly associated with the progression of GC (Yu et al., 2021). Non-T-cell inflammatory tumors exhibit a heightened enrichment of tumor-intrinsic Wnt/β-catenin signaling as identified in The Cancer Genome Atlas (TCGA). Tissues with activated Wnt/β-catenin signaling showed significantly reduced expression of T-cell inflammatory genes in non-T-cell inflammatory tumors compared to matched normal tissues (Luke et al., 2019), which results in immune rejection of the cancer and affects ICI therapy. A recent study revealed, high Wnt/β-catenin expression in certain types of GC cells is generally related to the striking loss of CD8 (+) T-cell infiltration, while the blockage of this pathway can inhibit their ability to migrate and invade other cells. They also found that Wnt/β-catenin downregulation may enhance the sensitivity of GC cells to PD-1 antibodies *in vitro* (Li et al., 2022). Another study found low levels of ISG12a, an innate immune effector that can suppress PD-L1 expression by affecting the canonical Wnt/β-catenin signaling pathway in gastrointestinal tumors, especially GC (Deng et al., 2020). Theoretically, increased expression of PD-L1 via aberrant Wnt/β-catenin pathway activation in GC might boost anti-PD-L1 therapy, but no relevant experiments have been reported. Taken together, aberrant Wnt/β-catenin pathway activation in GC may enhance immune evasion and resistance to ICI therapy, but the exact molecular mechanism is yet to be studied.

#### 2.1.3 Loss or mutation of immunoedited antigens

By establishing conditions in the tumor microenvironment (TME) that promote tumor growth, the immune system can not only inhibit tumors by suppressing their growth or killing cancer cells but also promote tumor progression, which is called cancer immune editing. There are three distinct stages in the current framework for cancer immune editing: elimination, balance, and evasion (Schreiber et al., 2011; Vesely and Schreiber, 2013). In the field of tumor immunology, a main advance has been the demonstration that cancer patient’ immune systems can react strongly to antigens expressed in their tumors by developing high levels of specific antibody and T cells (Schreiber et al., 2011). Immunoedited antigens on the surface (or inside) of tumors, such as PD-L1, are essential for tumor recognition and activation by immune cells (especially T cells). The loss or mutation of these antigens is likely to have implications for different types of immunotherapies that require corresponding antigenic targets, as supported by a study of a subset of GC patients (Li et al., 2022). Vesely et al. showed that a mechanism by which cancer cells evade immune detection is by immune selection for tumor variants lacking strong tumor-specific antigens (Vesely and Schreiber, 2013). Another study has also shown that as tumors grow they can acquire mutations and produce neoantigens that may affect the response of patients to ICIs. Subclonal neoantigens induced by cytotoxic chemotherapy have been shown to contribute to increasing tumor mutational burden (TMB) and are enriched in some poor responders (McGranahan et al., 2016). This suggests that loss or variation of neoantigens may affect immune surveillance and the efficacy of ICIs. In addition, TMB can influence the tumor response to ICIs by promoting a high immunogenic antigen load, as demonstrated in several experiments (Le et al., 2015; Goodman et al., 2017; Cristescu et al., 2018). The study by Kwon and others reported that increased microsatellite instability (MSI) and TMB were observed in 20% of GC patients and were associated with a clinical benefit with PD-1 antibody (Kwon et al., 2021). Similarly, a case report by Chen et al. showed that a GC patient with mismatch repair proficiency and microsatellite stability showed a definitive objective response to anti-PD-1 therapy treated with pembrolizumab (Chen et al., 2016). In conclusion, the results of multiple independent studies have shown that mutation or loss of mutation-associated neoantigens makes tumors resistant to anti-PD-1 therapy, but some markers may not be able to adequately predict anti-PD-1 therapy resistance in GC, and the relevant mechanisms and clinical experiments need to be further studied.

#### 2.1.4 Deficiency of tumor antigen presentation

Antigen presentation refers to the process in which antigens are taken up by antigen presenting cells (APCs), processed and presented on the cell surface in the form of major histocompatibility complex (MHC) peptide complexes, and finally recognized by T lymphocytes. To trigger an effective antitumor response, it not only requires dendritic cells (DCs) to take up the cancer cell surface antigen and cross-presented to activate CD8 T cells but also, the tumor with the antigen must be recognized accurately and thus killing by the T cells. In these two steps, tumors can evade immune recognition by exploiting a variety of escape mechanisms (Sánchez-Paulete et al., 2017; Jhunjhunwala et al., 2021). Since MHC-I molecules are not crucial for cell survival, the loss or defect of MHC-I antigen presentation is an important mechanism by which cancers evade immune system. This not only impairs the ability of the natural immune response to inhibit cancer but also affects the effectiveness of ICIs, which work by reactivating antitumor CD8 T cells (Dhatchinamoorthy et al., 2021). Therefore, increasing MHC-I expression in treatment can enhance ICI efficacy, which has been demonstrated in preclinical trials (Gu et al., 2021). Shukla et al. developed a computational pipeline capable of accurately inferring Class I HLA-A, B, and C germline alleles and using their inferred alleles as a reference to detect mutations in these genes. As a result, a total of 298 non-silent HLA mutations were identified in tumors from 266 patients based on whole-exome sequencing data from 7,930 pairs of tumor and healthy tissue from the same patient. Moreover, GC is more susceptible to HLA-I mutations than some other types of cancers such as ovarian cancer and chronic lymphocytic leukemia (Shukla et al., 2015). This demonstrates that there is a certain degree of HLA-I deficiency in tumor patients, and even in subtypes of the same cancer, there are clear differences. In a GC study, Iwasaki and others compared 58 MSI, 44 EBV-positive, and 107 non-EBV non-MSI tumors. The results showed that the frequency of HLA-I defects (≥1%) in MSI tumors was obviously higher (52%) than that in EBV-positive tumors (23%) and other tumors (28%). Additionally, HLA-I-deficient tumor areas had a significant lower levels of CD8 (+) cells infiltration than HLA-I-preserved tumor areas within the tumor (Iwasaki et al., 2021). I believe that these experimental results will leave adequate room for imagination about the effect of tumor antigen alteration and its presentation on ICIs.

Notably, APscore, a predictor of prognosis and response to ICIs, was developed by Wang and the team recently based on genes associated with antigen processing and presentation in advanced GC (Wang et al., 2022). This provides a new way of thinking to avoid the possible situation of ICI resistance. In summary, defects in tumor antigen presentation, such as the failure of MHC-I antigen presentation, may be a source of ICI resistance.

### 2.2 Tumor-extrinsic mechanisms

#### 2.2.1 T cell rejection

In the treatment of ICIs, T cells are undoubtedly one of the most critical players because killing tumors needs to be mediated by cancer-specific T cells. The full potential of T-cell-mediated tumor immunotherapy, including ICIs, will not be realized if T cells are rejected or have too little infiltration in the TME. Cancer cells in the TME are generally able to prevent T-cell infiltration to achieve T-cell rejection through a variety of mechanisms (Joyce and Fearon, 2015; Yu et al., 2022). One example is that tumor-secreted transforming growth factor-β (TGF-β) suppresses antitumor immunity by shaping the TME and limiting T-cell infiltration (Mariathasan et al., 2018). To test whether selective TGF-β inhibition is sufficient to overcome ICI resistance, Martin et al. utilized SRK-181, a fully human antibody with a strong affinity, to specifically bind to TGF-β and inhibit its activation. The results showed that a combination of the antibody and anti-PD-1 treatment led to an increase in intratumoral CD8 T-cell infiltration and a decrease in immunosuppressive myeloid cells (Martin et al., 2020). This implies that TGF-β signaling activity may serve as a potential intervention point to overcome ICI resistance, but whether targeting TGF-β to overcome ICI resistance will be successful in GC patients is uncertain and needs to be verified by relevant clinical trials. In another study, to predict the efficacy of ICIs for cancer treatment, Jiang and his team developed TIDE, a computational approach, which can model two major mechanisms of tumor immune evasion: preventing T-cell infiltration and inducing T-cell dysfunction in the TME. Furthermore, in large tumor cohorts, they examined how the expression of each gene interacted with cytotoxic T lymphocyte infiltration to characterize T-cell dysfunction. They also used the expression profile of immunosuppressive cells to mimic the factors by which the tumor ruled out T-cell infiltration (Jiang et al., 2018). To a certain extent, this can avoid the occurrence of resistance to first-line anti-PD-1 or anti-CTLA-4 therapy due to T-cell rejection, but it is not from enough to solve the fundamental problem of ICI resistance, and further clinical research is needed. Additionally, a recent study has shown that when the immune system attacks tumor cells, they will be close to each other to hide under the overlapping cell membranes, while T cells cannot achieve direct contact with them, resulting in immune escape of the inner tumor cells. More importantly, certain signals released by T cells can be detected by tumor cells, enabling them to recognize when the immune system will attack. Gutwillig et al. found some of these signals and revealed that blockade of these signals could prevent the tumor from escaping the immune cells, which is beneficial to immunotherapy (Gutwillig et al., 2022). Thus, this signaling pattern of cancer cells rejecting T-cell killing may lead to ICI resistance, and blocking the probe signal of the relevant tumor could enhance the immune response to ICIs.

#### 2.2.2 Immunosuppressive cells

**FIGURE 3:**
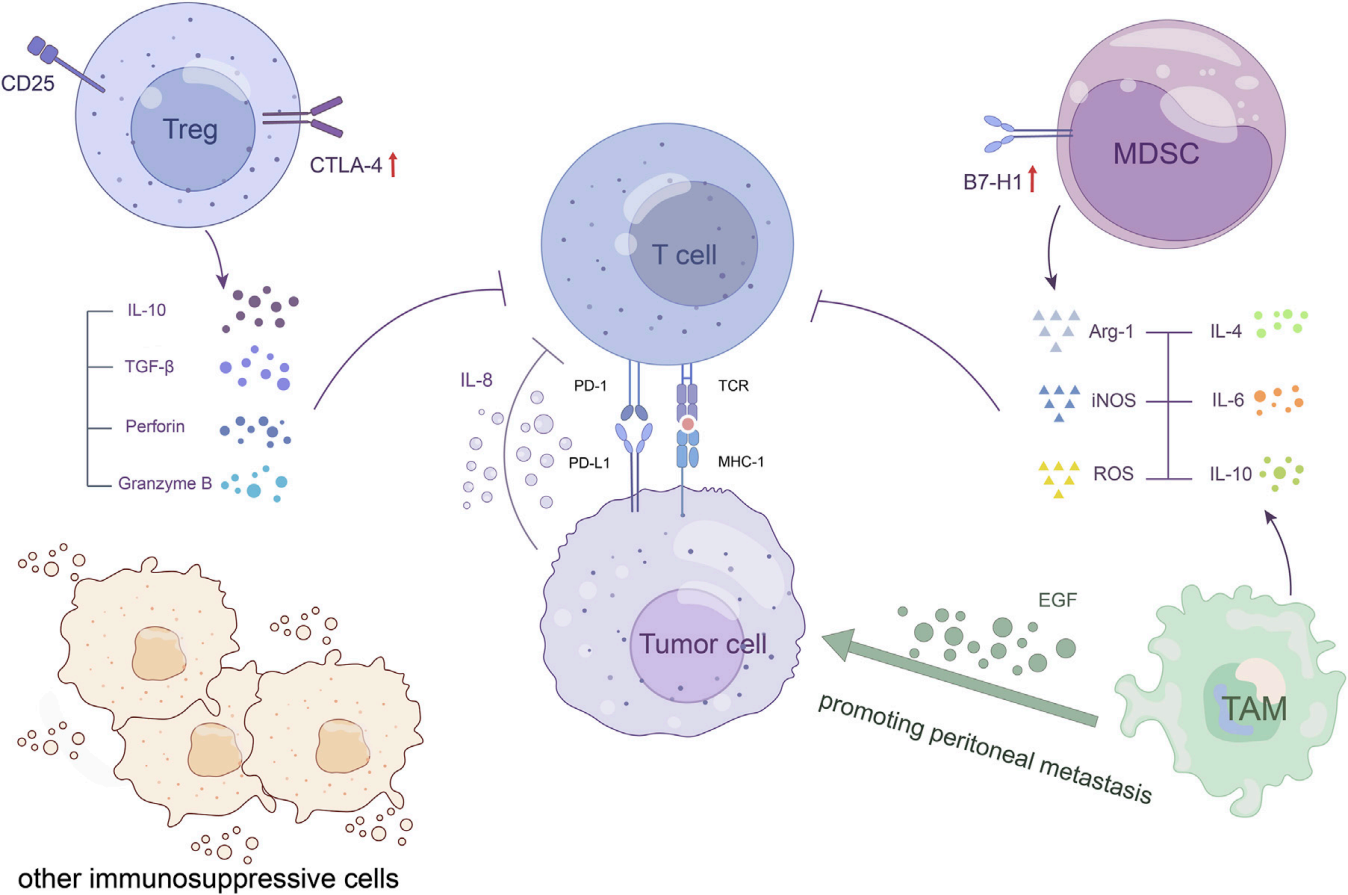
Immunosuppressive cells. Immunosuppressive cells within the TME establish cellular networks to suppress host immunity and promote tumor growth, which may affect the potential of ICI treatment. Tregs upregulate immune checkpoint molecules, such as CTLA-4, which block the activation of T cells, enhance the accumulation of Treg, and promote tumorigenesis. They also release granzyme B and perforin to induce cytolysis of effector T cells. MDSCs increase PD-L1 expression to induce T-cell anergy and induce Treg proliferation through cellular networks in the TME. TAMs promote peritoneal metastasis of GC through the epidermal growth factor receptor signalling pathway.

Immunosuppressive cells commonly found in the TME, such as regulatory T cells (Tregs), MDSCs, tumor-associated macrophages (TAMs), and other immunosuppressive cells, may affect ICIs through a variety of direct or indirect mechanisms (Figure 3). These include affecting the expression of molecules related to ICIs, reducing the number of infiltrating T cells and weakening T-cell function (Liu et al., 2022). Although there is no clear clinical evidence, we speculate that immunosuppressive cells are likely to be an essential factor in ICI resistance based on the results of preclinical experiments (Table 2).

**TABLE 2:** Factors and functions associated with induction of GC immune resistance in the TME.

Factors	Functions	References
PD-1/PD-L1	Inhibiting CD8 T cell activation/proliferation	Freeman et al. (2000), Dong et al. (2002)
	Reducing the production of proinflammatory cytokines	
CTLA-4/B7-1 or CTLA-4/B7-2	Delivering inhibitory signals to CD8 T cells	Brunet et al. (1987)
DCs	antigen presentation	Jhunjhunwala et al. (2021)
Tregs	Producing IL-10、TGF-β; upregulating immune checkpoint molecules, such as CTLA-4	Ichihara et al. (2003), Mao et al. (2017), Saleh and Elkord (2019)
	releasing granzyme B and perforin to induce cytolysis of Teffs	
	establishing cellular networks with other immunosuppressive cells within the TME	
Bregs	Producing IL-10、IL-35、TGF-β; expressing inhibitory molecules such as FasL and PD-L1	Jing et al. (2021)
MDSCs	increasing PD-L1 expression to induce T cell anergy	Veglia et al. (2018), Oya et al. (2020), Li et al. (2021)
	suppressing host immunity through Arg-1, iNOS and ROS	
	inducing Treg proliferation	
M1 macrophage	promoting inflammatory reaction through producing type I proinflammatory cytokines such as IL-1β, IL-1α, IL-12, TNF-α	Gambardella et al. (2020)
M2 macrophage	inhibiting inflammatory reaction through producing IL-4, IL-6, IL-10 and having pro-tumorigenic functions	Gambardella et al. (2020), Chen et al. (2021), Zhou et al. (2021)
	inducing angiogenesis	
	promoting peritoneal metastasis of GC	
CAFs	facilitating GC cell proliferation through CAF-derived IL-6	Kato et al. (2018), Sun et al. (2022)
	controling angiogenesis	
TANs	expressing high levels of immunosuppressive molecules FasL and PD-L1	Wang et al. (2017), Hiramatsu et al. (2020), Shan et al. (2022)
Mast cells	promoting GC cell proliferation, migration, invasion and inhibiting apoptosis	Zhong et al. (2018)
LAG-3	Inhibiting CD8 T cell activation/proliferation; enhancing the activity of Tregs in the TME	Anderson et al. (2016), Qin et al. (2019)
	promoting MDSCs	
TIM-3	inducing CD8 T cell exhaustion	Anderson et al. (2016)
IL-8	increasing angiogenesis	Schalper et al. (2020), Fousek et al. (2021), Li et al. (2022c)
	recruiting infiltrating immune suppressive cells, especially MDSCs and TAMs	
IL-10	Reducing the production of proinflammatory cytokines; inhibiting APCs; inhibiting memory Th17 and Th2 cells	Ouyang and O'Garra (2019)
	promoting the survival and activity of Tregs	
TGF-β	inhibiting Th1 and CD8 T cells	Batlle and Massagué (2019), Derynck et al. (2021)
	promoting differentiation of CD4 T cells to a Treg phenotype	
	inducing DCs dysfunction	

As we know, naturally occurring Tregs play an important role in the maintenance of immune self-tolerance and immune homeostasis (Wing et al., 2008). However, some Tregs are also one of the main types of tumor-infiltrating immune cells, which are able to establish cellular networks with other immunosuppressive cells within the TME and have strong immunosuppressive properties (Lucca and Dominguez-Villar, 2020). In preclinical models of cancer, it has been found that CD25 molecules are expressed at high levels on Tregs, and the precise targeting of CD25 Tregs combined with anti-PD-1 treatment works synergistically (Arce Vargas et al., 2017). This partially demonstrates the negative effect of Tregs in the TME on ICI treatment. Tregs in the TME have been found to have upregulated levels of immune checkpoints/coinhibitory receptors, such as CTLA-4, which can block T-cell activation, enhance the accumulation of themselves, and promote tumorigenesis (Saleh and Elkord, 2019). Another study reported that GC patients showed higher Treg infiltration and advanced disease progression as well as reduced survival (Ichihara et al., 2003; Mao et al., 2017). These tumor-associated Tregs exerted significant immunosuppressive properties and suppressed CD8 T-cell immune functions, including the production of granzyme B and CD8 T-cell proliferation *in vitro*. They also promote GC cell growth and development through IL-10 secretion and cell‒cell contact mechanisms (Mao et al., 2017), which may reduce the effect of ICI treatment.

MDSCs are heterogeneous cell populations arising from many pathological conditions from inflammation to cancer. These cells inhibit the function of T cells and reduce the efficacy of antitumor immunity (Veglia et al., 2018; Oya et al., 2020; Li et al., 2021). Using a preclinical mouse model system, De Henau and others showed that ICI resistance is closely associated with the inhibitory activity of infiltrating myeloid cells in several tumors. Targeting selectively the phosphoinositide 3-kinase gamma (PI3Kγ), which is highly expressed in myeloid cells, can restore sensitivity to ICIs (De Henau et al., 2016). The study mentioned above revealed that PMN-MDSC frequency but not M-MDSC frequency is related to survival in GC patients. In addition, the CXCR2 antagonist can reduce PMN-MDSC accumulation, increase CD8 T cell infiltration in the tumor, and further enhance the efficacy of anti-PD-1 in tumor-bearing mice (Zhou et al., 2022). This shows the difference in different MDSC subtypes in tumor immunosuppressive effects and the enhancement of ICI efficacy by inhibiting MDSCs, highlighting the role of MDSCs in ICI resistance. In conclusion, there is increasing evidence that GC patients who show a high level of MDSC infiltration have a relatively poor prognosis and are more likely to be resistant to ICIs (Gabitass et al., 2011; Zhuang et al., 2012; Kim et al., 2021).

TAMs can promote tumor cell proliferation, angiogenesis and immunosuppression, resulting in tumor progression, metastasis and drug resistance (Gambardella et al., 2020; Chen et al., 2021; Zhou et al., 2021). Recently, it has been shown that immunosuppressive macrophages promote tumor immune escape and impede anti-PD-1 therapy in preclinical GC models *in vitro* and *in vivo* (Shi et al., 2022). In one study, a set of data from 298 GC patients from TCGA suggested that IL-10+ TAM infiltration generates an immune evasion TME characterized by Treg infiltration and CD8 (+) T-cell dysfunction (Zhang et al., 2022). The above experiments to some extent revealed the correlation between TAMs and resistance to ICI treatment in GC. However, more studies have shown that high infiltration of TAMs can lead to an increase of PD-L1 expression on tumor cells (Harada et al., 2018; Lin et al., 2019; Yagi et al., 2019), thereby enhancing the sensitivity of tumors to anti-PD-L1 in theory. This contradiction may be that the effect of high expression of tumor PD-L1 in the treatment of ICIs is not enough to offset the other immunosuppressive effects of TAMs in the complex TME. Therefore, research on relevant mechanisms needs to be carried out. Currently, targeting these immunosuppressive cells is a major focus of clinical research and is also considered a promising strategy to overcome ICI resistance (Mantovani et al., 2017).

Other cells that exert immunosuppressive functions in the TME, such as cancer-associated fibroblasts (CAFs) and tumor-associated neutrophils (TANs), have similar immunosuppressive mechanisms and can also affect the potential of ICI therapy in GC patients (Wang et al., 2017; Kato et al., 2018; Hiramatsu et al., 2020; Shan et al., 2022; Sun et al., 2022).

#### 2.2.3 Inhibitory factors in the TME

Inhibitory molecules in the TME, including various novel immune checkpoint molecules that have been identified, such as lymphocyte-activation gene 3 (LAG-3), T-cell immunoglobulin and mucin domain-containing molecule 3 (TIM-3) (Anderson et al., 2016; Qin et al., 2019), some cytokines and immunosuppressive factors, such as IL-8 (Schalper et al., 2020; Fousek et al., 2021; Li et al., 2022), IL-10 (Ouyang and O'Garra, 2019), and TGF-β (Batlle and Massagué, 2019; Derynck et al., 2021), can inhibit host immune function through direct or indirect pathways, leading to tumor growth and immune escape. However, the extent to which these inhibitory molecules contribute to resistance to ICI therapy in cancers, particularly in GC, remains largely poorly understood owing to a lack of clinical data. Here, we briefly discuss the inhibitory molecules in GC that have a high likelihood of having an impact on ICI resistance (Table 2).

LAG-3 is an immunosuppressive receptor, and MHC-II is considered to be its typical ligand (Maruhashi et al., 2022). The blockade of one of PD-1, LAG-3, and CTLA-4 showed compensatory upregulation of other immune checkpoint molecules, enhancing their ability to suppress local T cells in a tumor model study (Huang et al., 2017). In addition, Woo et al. revealed that LAG-3 and PD-1 were widely coexpressed on CD8 and CD4 T cells in three different transplantable tumors, and they synergistically regulated T-cell function and promoted tumor progression (Woo et al., 2012). This suggests that when anti-PD-1, anti-CTLA-4 or anti-PD-1/anti-CTLA-4 in combination is used alone, tumor cells can develop immune resistance through compensatory upregulation of the immunosuppressive molecule LAG-3 and reduce the therapeutic effect. These data show promising prospects for LAG-3 blockade to alleviate resistance to ICI treatment, such as anti-PD-1 therapy. Multiple strategies of LAG-3 antibodies combined with other immunotherapies are being implemented (Maruhashi et al., 2020). TIM-3, a transmembrane protein, is the member of the TIM gene family (Acharya et al., 2020). In a mouse model of cancer that progressed after anti-PD-1 treatment, TIM-3 was found to be upregulated in the antibody-bound T cells. The blockade of TIM-3 after PD-1 blocking failed showed an improved survival. At the same time, data revealed that some tumor patients developing immune resistance to anti-PD-1 therapy showed a similar upregulation of TIM-3 when the treatment failed (Koyama et al., 2016). This suggests that TIM-3 may play a role in the resistance of ICI treatment. Previous studies have showed that IL-8 may be an independent and essential prognostic factor in GC patients (Kido et al., 2001). A large retrospective analysis also revealed that some advanced tumor patients treated with ICIs had an elevated baseline serum IL-8 level which was associated with poor prognosis. This further shows the importance of IL-8 as an independent biomarker for patients with associated tumors (Schalper et al., 2020). The finding also implies that elevated serum IL-8 may be connected with ICI treatment resistance in GC patients, although the mechanism involved remains unclear. In addition, a study recently found that tumor-derived IL-8 upregulates PD-1 in CD8 T cells, which promotes lymph node metastasis of GC (Li et al., 2022). This may be the result of negative feedback mechanisms of autoregulation. Overall, despite some encouraging results, the current evidence is still insufficient to determine the extent to which upregulation of immune checkpoint molecules contributes to anti-PD-1 or anti-CTLA-4 resistance in GC patients. The mechanism by which IL-8 and other cytokines are involved in ICI resistance is also not completely understood. Therefore, more relevant studies need to be carried out.

#### 2.2.4 Microbiota

As an integral part of the human body, the microbiota not only provides assistance for immune function but also contributes to homeostatic immunity (Rooks and Garrett, 2016; Belkaid and Harrison, 2017). Meanwhile, the microbiota and its metabolites are thought to have systemic and local effects on tumor onset, progression, and response to immunotherapy (Elinav et al., 2019). As ICIs become the forefront in the development of immunotherapy, the role of microbiota in ICI immunotherapy has become a research hotspot as well. In the following, we will focus on the possible involvement of the microbiota and its metabolites in ICI resistance in GC patients.

In microbiota, *H. pylori* infection is closely related to GC (Lee et al., 2016). Recently, 34 H. pylori-positive patients (44.2%) in 77 advanced GC patients treated with PD-1 antibody were found to have a higher risk of nonclinical response to anti-PD-1 therapy than H. pylori-negative patients, with an OR of 2.91 (95% CI: 1.13–7.50) (Che et al., 2022). Some patients with other types of tumors also showed similar results (Oster et al., 2022). This suggests that *H. pylori* infection may affect the function of ICIs through some as-yet-unelucidated mechanism, thereby reducing the survival time of patients treated with ICIs. In preclinical and clinical studies, multiple lines of evidence have gradually demonstrated that the gut microbiota influences antitumor immunity and ICI immunotherapy efficacy through pathways such as metabolites (Lu et al., 2022). For example, Mirji et al. recently identified trimethylamine N-oxide (TMAO), a gut microbiota-derived metabolite, and found that it can enhance the type I IFN pathway and thus exert antitumor effects. Moreover, in a mouse model, coadministration of TMAO with ICIs (anti-PD-1 and/or anti-TIM-3) can obviously reduce tumor burden and improve survival patients compared with TMAO or ICIs alone (Mirji et al., 2022). At present, many studies have revealed that the diversity of the gut microbiome is closely related to a good response to ICIs in tumor patients (Gopalakrishnan et al., 2018; Jin et al., 2019; Mager et al., 2020), and some species may become potential biomarkers for improving patient stratification in future immunotherapy studies (Lee et al., 2021; Derosa et al., 2022). Therefore, intestinal dysbiosis, such as a lack of certain bacterial species, may be one of the causes of ICI resistance in GC patients, which would impair the response to ICI treatment. This insight is supported by data from multiple clinical studies (Derosa et al., 2018; Pinato et al., 2019; Tinsley et al., 2020). Additionally, clinical studies also revealed that patients with advanced cancer who receive antibiotics have a shorter survival time than similar patients who do not receive antibiotics. More importantly, the clinical benefit of ICIs in these patients is reduced. In contrast, fecal microbiota transplantation (FMT) can help overcome resistance to anti-PD-1 therapy in patients with refractory cancer (Routy et al., 2018; Baruch et al., 2021; Davar et al., 2021). Therefore, under certain conditions, antibiotics should be prescribed with caution to these patients treated with ICIs. Interestingly, FMT has also been suggested to help address immune-related adverse events (irAEs), such as treatment-refractory ICI-associated colitis (Wang et al., 2018), which to some extent shows the suppression of the potent toxic effects of ICIs by the microbiota. Considering the unique microbial ecological environment in the gastrointestinal tract and the current research results, we have reason to believe that the microbiota will play an important role in ICI resistance in GC patients.

## 3 Other mechanisms

### 3.1 Metabolism

In recent years, it is believed that metabolism in the TME may contribute to ICI resistance. Since the discovery of the Warburg effect in the last century, the role of cancer cell metabolism in maintaining its occurrence, progression, metastasis and drug resistance has been largely clarified (Icard et al., 2018; Martínez-Reyes and Chandel, 2021). In contrast, metabolic reprogramming of immune cells (especially T cells) has been less studied, and this process is thought to be one of the mechanisms that can promote antitumor immunity and enhance ICI efficacy (Biswas, 2015). For example, in a preclinical study, tumor-specific CD4 and CD8 T cells were found to metabolically reprogram phosphoenolpyruvate carboxylation kinase 1 (PCK1) by increasing the glycolytic metabolite phosphoenolpyruvate (PEP) to enhance the antitumor response (Ho et al., 2015). Similarly, in several other similar studies, it has been found that the antitumor activity and survival of T cells are enhanced by modulating selective amino acid and cholesterol metabolic changes (Geiger et al., 2016; Yang et al., 2016; Leone et al., 2019), thereby enhancing ICI responses. Thus, dysregulation of immune metabolism may reduce the rate of response to ICIs and produce GC immune resistance. In addition, the role of inflammation, hypoxia, acidity and other factors related to cell metabolism in the TME in immunotherapy resistance has also been elucidated with the progress of related research (Huber et al., 2017; Wattenberg and Beatty, 2020; Kopecka et al., 2021). For exmaple, lactic acid in the TME can inhibit immune cells and promote immunosuppressive cells to hinder anti-tumour immunity directly or indirectly (Hayes et al., 2021). In GC, increased lactic acid level was correlated negatively with percentages of Th1 cells and cytotoxic T lymphocytes (Ping et al., 2018), which may lead to ICI resistance. However, whether metabolism can cause immune resistance to GC treatment still lacks sufficient evidence, and some therapies targeting related metabolic pathways combined with ICI treatment are currently being tested in clinical trials (Kraehenbuehl et al., 2022).

### 3.2 Epigenetics

The process of heritable changes in a gene’ function without altering its DNA sequence of is called epigenetics. By driving aberrant transcriptional programs, dysregulation of the epigenome affects tumor immunogenicity and the function of immune cells involved in the TME and promotes tumor growth and progression, potentially leading to ICI immune escape or resistance (Hogg et al., 2020). For example, tumors with high alternative promoter burden (APB) were found to have little human T-cell infiltration in a preclinical model of GC. Meanwhile, data from patients with gastrointestinal cancer who received immunotherapy showed that APB-high tumors were more resistant to ICIs (Sundar et al., 2022). Mechanistically, changes in the epigenetic promoter region allow tumors to utilize alternative transcriptional start sites and to reduce expression of immunogenic N-terminal peptides, which ultimately leads to ICI resistance (Sundar et al., 2019). Scott et al. found that the nuclear factor TOX is a key regulator of tumor-specific T-cell differentiation. *In vitro*, experiments have shown that a transcriptional program associated with T-cell depletion may be activated when the expression of TOX becomes abnormal in effector T cells. Furthermore, deletion of TOX in the T cells disabled the original depletion program, as tumor-specific T cells lacking TOX no longer upregulated genes for inhibitory receptors. However, to the scientists’ surprise, the TOX-deleted T cells with a lack of PD-1 and other inhibitory molecules remained dysfunctional and did not survive for a long period (Scott et al., 2019), which can impair ICI therapy. A study has shown that the SWI/SNF chromatin remodeling complex, a chromatin regulator, is associated with immune resistance. The response of tumor cells with the mutated genes to ICIs is stronger (Pan et al., 2018). In addition, recent studies have shown that noncoding RNA (ncRNA), as a key player in epigenetic regulation, can play a potential role in ICI resistance by directly or indirectly regulating genes involved in immunoregulation (Vishnubalaji et al., 2020). These studies suggest that the mechanisms of ICI resistance may involve deeper, epigenetically driven programs. Finally, although there is currently no clear evidence for epigenetics as one of the mechanisms of ICI resistance in GC, the strategy of combining selective epigenetic modifiers or inhibitors with immunotherapy is ongoing and has shown some benefit in some clinical and preclinical studies (Zhang et al., 2018; Fang et al., 2021; Kumar et al., 2021; Micevic et al., 2022).

## 4 Conclusion and prospects

ICIs play an important role in the field of tumor immunotherapy and bring hope to a considerable number of patients with advanced malignant tumors. However, most patients with advanced GC do not have satisfactory clinical benefit due to treatment ineffectiveness or strong resistance to ICIs. The combination of some chemotherapeutic agents, such as platinum, fluorouracil and taxane, and surgical treatment are still the standard options for the treatment of GC patients. At present, the complex mechanisms of resistance to ICIs remain largely unknown, which greatly limits their therapeutic potential. Therefore, this review focuses on summarizing the mechanisms of resistance to ICI immunotherapy in GC, to provide a better understanding for related research.

ICIs have shown encouraging efficacy in some tumors, paving the way for their wider development. However, in GC, our understanding of the complex resistance mechanisms of ICIs is still in its infancy, and some questions remain. First, ICIs can only induce longer-lasting antitumor responses in a few subsets of advanced GC, and the efficacy of single-agent therapy is limited. The extent to which resistance mechanisms to ICIs are consistent across different GC cell subsets is not clear, nor can it be determined which types of GC cells have specific resistance mechanisms. Second, the current research on GC immunotherapy resistance is significantly less than that on other cancers, such as melanoma and lung cancer. Some of the resistance mechanisms mentioned above have not been confirmed in GC, and some animal research results lack the support of corresponding clinical data, which hinders the application of ICIs in GC patients. Additionally, there is still a lack of clinical evidence on whether the unique physicochemical environment in the stomach, such as digestive juice, the type of food ingested, and the intestinal microbiota mentioned in this review, is related to ICI resistance. The combination of immunotherapy with other standard treatments such as chemotherapy and radiotherapy has been used to overcome resistance to ICIs. New strategies will involve the combination of drug usage, the combination of ICIs and CAR-T therapy, and the search for the biomarkers for ICI resistance. In the future, to more fully understand the resistance mechanism of ICIs in GC patients, apart from solving some of the above problems, it is also necessary to give attention to the new discoveries of resistance mechanisms in some well-studied cancers and transfer them to the background of GC for further research to lay a foundation for achieving better clinical efficacy of ICIs in GC.

## Figures and Tables

**Table:** 

**ICI**	immune checkpoint inhibitor
**B7-H1**	B7 homolog 1
**PD-L1**	programmed death ligand 1
**MAPK**	mitogen-activated protein kinase
**Treg**	regulatory T cell
**Breg**	regulatory B cell
**MDSC**	myeloid-derived suppressor cell
**TAM**	tumor-associated macrophage
**TME**	tumor microenvironment
**LAG-3**	lymphocyte-activation gene 3
**TIM-3**	T cell immunoglobulin and mucin domain–containing molecule 3
**IL**	interleukin
**TGF-β**	transforming growth factor-β
**TCR**	T cell receptor
**DC**	dendritic cell
**MHC**	major histocompatibility complex
**JAK**	Janus kinase
**STAT**	signal transducer and activator of transcription
**MSI**	microsatellite instability
**TMB**	tumor mutational burden
**TMAO**	trimethylamine N-oxide
**GC**	gastric cancer
**IFNGR**	IFN-γ receptor
**APC**	antigen presenting cell
**TAA**	tumor-associated antigen
**CTLA-4**	cytotoxic T lymphocyte-associated antigen-4
**ARG-1**	arginase-1
**EGF**	epidermal growth factor
**iNOS**	inducible nitric-oxide synthase
**ROS**	reactive oxygen species
**Teff**	effector T cell
